# Long-Term Benefits of Photobiomodulation Therapy on Health-Related Quality of Life in Burning Mouth Syndrome Patients: A Prospective Study

**DOI:** 10.3390/jcm13144272

**Published:** 2024-07-22

**Authors:** João Mendes de Abreu, Tiago Nunes, Pedro A. Almiro, José Figueiredo, Ana Corte-Real

**Affiliations:** 1 Faculty of Medicine, University of Coimbra, 3000-548 Coimbra, Portugal; 2 Stomatology Service, Head, Neck & Skin Surgery Department, Coimbra Hospital and University Centre, 3004-561 Coimbra, Portugal; 3 Clinical and Academic Centre of Coimbra, 3004-561 Coimbra, Portugal; 4 Forensic Dentistry Laboratory, Faculty of Medicine, University of Coimbra, 3000-548 Coimbra, Portugal; 5 Research Centre for Psychology, Autonomous University, 1169-023 Lisbon, Portugal

**Keywords:** low-level light therapy, photobiomodulation, burning mouth syndrome, quality of life, health-related quality of life

## Abstract

**Background/Objectives**: Burning Mouth Syndrome (BMS) patients experience a reduction in health-related quality of life and an increased intake of medication. Photobiomodulation with low-level laser therapy has been demonstrated to be an efficacious treatment for BMS. However, its long-term benefits remain relatively unknown. This study aimed to evaluate the impact of prolonged Photobiomodulation with low-level laser therapy on BMS patients by examining the efficacy of an outpatient protocol in a real-world setting. **Methods**: A prospective study was designed to address the research question. Photobiomodulation was performed, irradiating the affected areas once every two weeks for 12 months. Health-related quality of life was assessed using the EQ-5D-5L questionnaire at the initial consultation and after 6 months and 12 months of treatment. Additionally, the patients’ pharmacological profile was also monitored. Nonparametric statistical analysis was performed (*p* < 0.05 was considered statistically significant). **Results**: The study was completed by 15 individuals, comprising 14 females and 1 male. The results indicated a statistically significant improvement (*p* < 0.001) in four of the five dimensions of the health-related quality of life questionnaire, namely self-care, usual activities, pain/discomfort, and anxiety/depression, along with the patients’ perceived health level. A total of 13 participants reported suspending or reducing their intake of medications for Burning Mouth Syndrome. **Conclusions**: Therefore, Photobiomodulation with low-level laser therapy has a positive effect on improving patients’ quality of life and reducing BMS symptoms, contributing to a subsequent reduction or suspension of previous medications. These findings support the efficacy of the applied protocol. Given the innovative methodology and promising results, further research is warranted.

## 1. Introduction

Burning Mouth Syndrome (BMS) is a condition characterized by a persistent intraoral burning sensation of varying intensity, occurring without visible lesions [1]. However, several different definitions have emerged in the literature [2,3]. Due to the significant variability in characterizing BMS, in 2020, the International Headache Society (IHS) sought to achieve consensus when producing the International Classification of Orofacial Pain (ICOP). This classification included the addition of novel symptomatology (such as dysesthesia or pain) along with a chronological setting (recurring daily for more than two hours a day for over three months) and noted the absence of specific laboratory findings [4].

It is estimated that the global prevalence of BMS can reach 1.73%, with a higher prevalence among the European population (5.58%). It is more prevalent in women, with studies suggesting a ratio as disparate as 3:1 to 7:1, and tends to occur more frequently between the fifth and seventh decades of life [5,6].

Several local and systemic factors have been identified as potential contributors to the development of BMS [7]. Still, recent studies have demonstrated the involvement of the nervous system at various levels in patients with BMS, accompanied by peripheral small fiber neuropathy and central functional magnetic resonance imaging alterations [6,8,9].

Nevertheless, the onset of BMS is typically spontaneous, although some patients tend to associate the emergence of the disease with specific events such as dental procedures, the commencement of medications, or stressful life events [10].

As the tongue is the most frequently affected site of the burning symptoms, patients can also present with analogous pain or discomfort in other areas of the oral cavity, which usually manifests bilaterally [6].

Furthermore, individuals with BMS frequently exhibit a higher prevalence of comorbidities, which is associated with a higher intake of medications and consequently results in a more compromised general health status [9,11] and a diminished quality of life (QoL) [6,12]. Mood swings and modifications in eating habits, as well as the onset of depression and a reduction in the desire to socialize, are also common changes experienced by patients with BMS [13].

The diagnosis of primary BMS represents a significant clinical challenge [9], with numerous BMS patients experiencing delays in diagnosis despite seeking and receiving professional care [14].

The aforementioned challenges also exert a significant impact on the management and treatment of these patients [3,14]. This has led to a situation in which many of the commonly implemented therapeutic strategies lack sufficient evidence to support or refute them. Clonazepam is the most commonly prescribed BMS drug, although its benefits are not unanimously accepted and remain a topic of debate regarding the optimal formulation and dosage [15,16].

Low-level laser therapy (LLLT) and/or Photobiomodulation (PBM) have demonstrated efficacy in the treatment of BMS, offering symptom reduction and improved quality of life. Furthermore, they present viable options as initial or second-line interventions [16,17,18,19,20,21,22,23]. These effects have been attributed to the analgesic, anti-inflammatory, and biological stimulation properties induced by tissue irradiation with infrared or near-infrared light, and thus the technique is a promising therapy, not only for BMS but also for other conditions characterized by acute or chronic pain [21,23]. Although the precise mechanism by which these effects are elicited remains uncertain, they are believed to emerge from intra- and extracellular changes, including augmented adenosine triphosphate (ATP) synthesis, serotonin production, and β-endorphin release. Additionally, there is a notable reduction in the firing of C-fiber neurons and a decrease in bradykinin secretion [17,18,22]. Nevertheless, given the considerable diversity in methodologies and substantial variations in laser parameters across the available studies, multiple systematic reviews dedicated to the topic have reached the same conclusion, underscoring the necessity for further clinical trials and robust evidence to establish their efficacy definitively [16,17,18,19,20,21,22,23].

In fact, none of the studies included in the literature address or analyze the effects of long-term PBM via LLLT in BMS patients. Moreover, the treatment protocols include PBM sessions on a weekly basis, or on multiple occasions within the same week, with the evaluation periods not exceeding the termination of the trial or a few months after [16,17,18,19,20,21,22,23]. Another unmeasured gap is the reduction or suspension of previous BMS-specific medication, which serves as a means of evaluating the perceived efficacy of PBM in decreasing or eliminating BMS symptomatology.

Therefore, the main objective of this study is to assess the effects of long-term PBM with LLLT on BMS patients by analyzing the efficacy of a real-life protocol, overcoming the previous shortcomings. This involves measuring the impact on health-related QoL (HRQoL) using the EQ-5D-5L questionnaire after 6 and 12 months while monitoring and managing the patients’ pharmacological profiles and considering the null hypothesis that the application of PBM therapy does not produce benefits in patients with BMS.

## 2. Materials and Methods

### 2.1. Study Desing and Institutional Approval

A prospective study was designed to address the following research questions: can the application of PBM therapy improve the HRQoL of patients with BMS? How can we manage patients previously treated with BMS-specific medication?

Prior to commencement, institutional approval was obtained from the Committee of the Faculty of Medicine of the University of Coimbra, Portugal (reference number CE-028/2022). All patients who participated in this study were presented with and required to sign an informed consent form.

All procedures were carried out in compliance with the principles outlined in the Declaration of Helsinki by the World Medical Association [24].

### 2.2. Patient Selection (Figure 1)

The inclusion criteria encompassed adult patients experiencing a persistent and recurrent intraoral burning sensation, with or without dysesthesia or pain. In addition, patients were required to display no visible lesions or relevant laboratory findings following the ICOP-1st ed definition of BMS [4]. The study population was recruited from the Dentistry and Stomatology Outpatient Departments at the Clinical and Academic Centre of Coimbra between 1 July 2022 and 31 December 2023.

Patients were excluded from the study if they were pregnant, had conditions that could exacerbate BMS symptoms (e.g., depression, fibromyalgia, smoking), or were suspected of having drug-associated xerostomia, which could confound the evaluation of HRQoL.

The inability to comprehend the text of the informed consent form and the questionnaire, as well as to comply with the appointment schedule, were also considered exclusion criteria.

**Figure 1:**
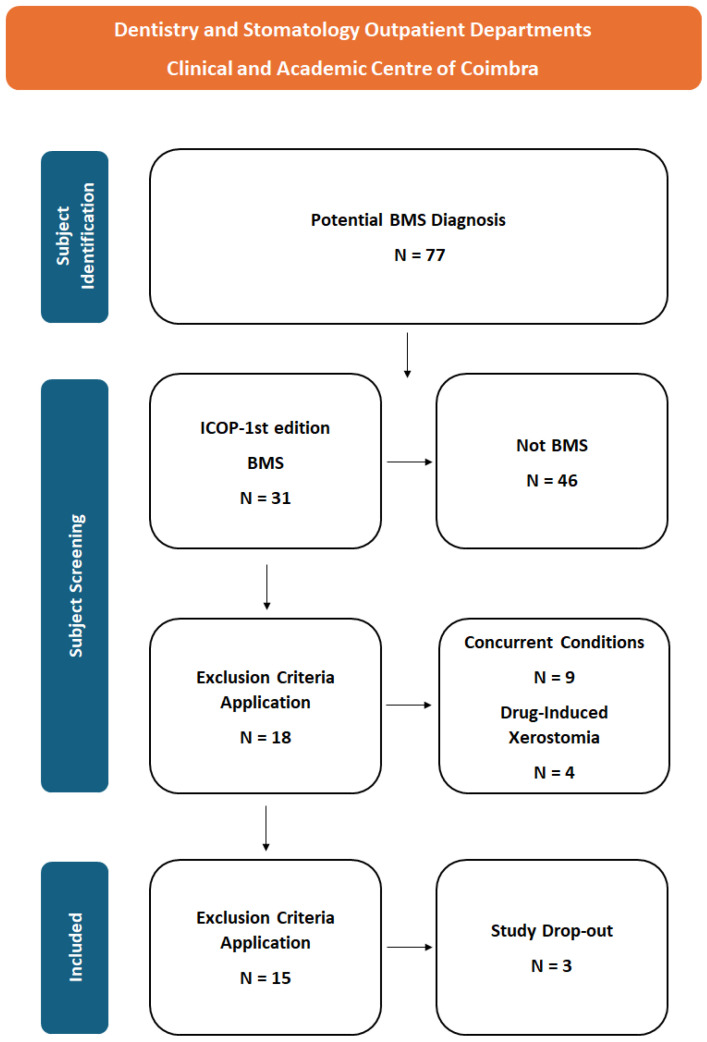
Patient selection flowchart.

### 2.3. Intervention and Conditions (Figure 2)

Oral PBM was conducted via the irradiation of the affected regions at two-week intervals over a 12-month period. This was achieved through the utilization of an LLLT device, specifically the Therapy XT^®^ (DMC, São Carlos, SP, Brazil), which employed pre-established parameters (continuous mode, λ = 660 nm, InGaAlP semiconductor, and a power output of 100 mW).

Throughout the course of the treatment, the probe remained in contact with the tissue, traversing a centimetric grid over the various sites where BMS symptoms were present, with a dosage of 6 J/cm² (60 s).

The treatments were conducted by the principal investigator, who wore appropriate protective eyewear during the LLLT. All safety procedures were followed.

The assessment of HRQoL was conducted using the EQ-5D-5L questionnaire in Portuguese [25] at three different stages: before the initial consultation, after 6 months, and following 12 months of treatment.

An 11-point Likert scale was employed to assess treatment satisfaction and the likelihood of recommending the treatment after a 12-month period.

Patients were encouraged and assisted in suspending or reducing current drug prescriptions if they were asymptomatic or experiencing mild symptoms. Furthermore, the patient’s pharmacological profile was evaluated after 12 months.

**Figure 2:**
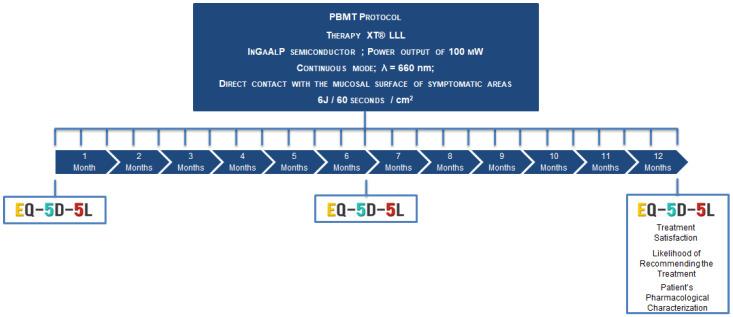
PBM experimental protocol.

### 2.4. Outcome Assessment and Variables

The EQ-5D-5L HRQoL questionnaire is a 5-dimensional, 5-level (1 to 5) assessment tool, comprising a visual analog scale (VAS) with a range of 0 to 100. The questionnaire evaluates patients’ capacity in terms of mobility, self-care, and usual activities, as well as assessing their level of pain/discomfort and anxiety/depression. Additionally, it includes the patients’ perception of their general health status, quantified on a scale from 0 to 100, with higher numbers representing better health.

The satisfaction with the treatment and the likelihood of recommending it were evaluated using an 11-point Likert scale, with higher scores indicating a more favorable outcome.

Other variables that were taken into consideration included sex, age, the affected areas (including the anatomical site and the number of areas affected), the characterization of the symptoms, the functional complaints, the time since the initial onset of the symptoms, the intake of BMS-specific prescriptions, and the reduction or suspension of said prescriptions.

### 2.5. Data Analysis

To ensure confidentiality, patient identities were concealed using alphanumeric anonymization. Furthermore, the results were assessed independently, without the involvement of the main researcher who conducted the clinical intervention.

Descriptive statistics were used considering the adequate statistical parameters.

The normality of the quantitative variables was assessed using Kolmogorov–Smirnov and Shapiro–Wilk tests.

Due to the sample size and variable distribution, nonparametric tests were applied. Inferential statistics were performed using the Friedman test and Spearman’s Rho to analyze the evolution and relation between study variables. A *p*-value of less than 0.05 was considered statistically significant.

A statistical study was performed using IBM SPSS Statistics^®^ software (Version 28.0.1.0 (142)).

## 3. Results

This study was completed by 15 patients, comprising an initial cohort of 18 participants. Of these, 14 were female and 1 was male. Most participants (46.7%) were in their seventh decade of life, with an average age of 69.53 years (±11.71), ranging from 37 to 80 years old. The mean time for the onset of initial BMS symptoms was 21.53 months (±14.5), with a range of 10 to 60 months. Of the 15 participants, 14 (93.3%) experienced symptoms for over a year. Among the excluded individuals, two failed to adhere to the predetermined appointment schedule, while one withdrew from the study for reasons unknown.

All patients identified the tongue as the primary site for BMS symptoms. Four patients (26.7%) reported symptoms in two different areas, while an additional four patients experienced symptoms in three distinct locations. The buccal region was the second most affected site, accounting for six cases (40%), followed by the hard palate with three cases (20%).

The most frequently reported symptom, cited by 14 patients (93.3%) as the primary reason for seeking medical treatment, was a burning sensation. Furthermore, four patients (26.7%) reported xerostomia as a concurrent complaint, while three (20%) mentioned dysgeusia. The prevalence of functional complaints was high, with 13 patients (86.67%) indicating at least one major limitation or inconvenience. The most common complaint was dietary restrictions, reported by 12 patients (80%), followed by the necessity of adopting soft- or neutral-flavor toothpaste, which was mentioned by 6 patients (40%).

### 3.1. Health-Related Quality of Life

The results demonstrated a statistically significant improvement in patients’ HRQoL across the three assessment points in four dimensions (self-care, usual activities, pain/discomfort, and anxiety/depression) of the EQ-5D-5L questionnaire. However, the fifth dimension, pertaining to patients’ mobility, remained unchanged (Figure 1) (Table 1). The effect size calculations for the Friedman test were achieved through the Kendall W test. The effect size is shown in Table 1.

**Figure 3:**
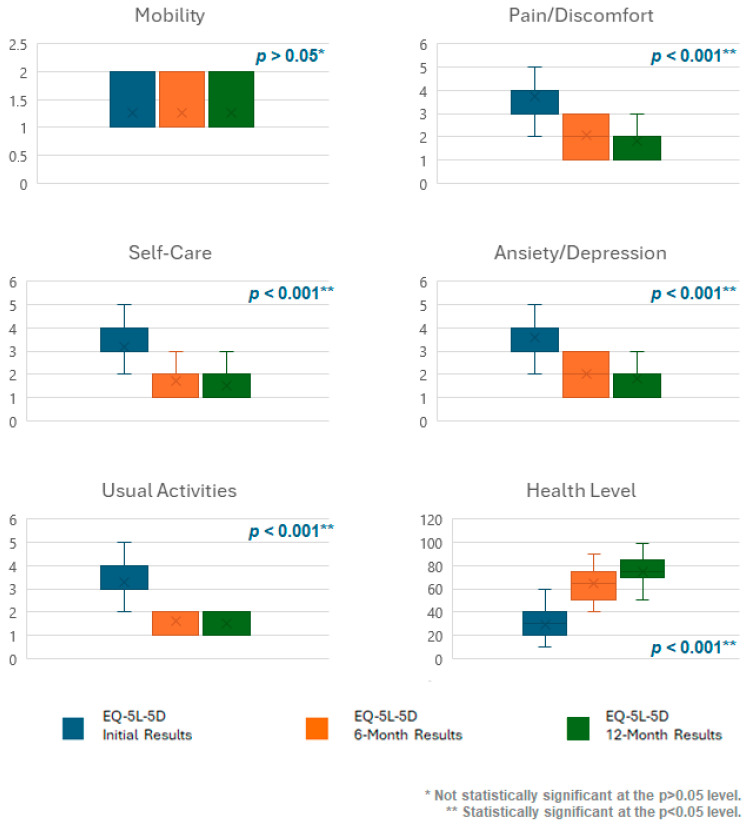
EQ-5D-5L questionnaire results before treatment, after 6 months, and after 12 months.

Furthermore, patients’ self-perceived health status, as indicated by the EQ-VAS, also demonstrated a statistically significant development across the three assessment points, with a mean score increase of 45.27 points from the beginning to the end of the study (Figure 3).

These results demonstrate statistically significant differences (*p* < 0.001) as determined by Friedman’s test for k paired samples (Table 1).

Additionally, Spearman’s Rho (ρ) yielded multiple statistically significant results, indicating a robust correlation between all four enhanced dimensions, the VAS score, and treatment satisfaction at the conclusion of the study (Table 2).

### 3.2. Pharmacological Characterization and Regulation of BMS-Specific Prescriptions

All patients reported taking prescription drugs for BMS, with eight participants (53.3%) having two or more different medications in their medical histories. Furthermore, the results demonstrated that four patients (26.7%) had previously attempted three different pharmacological agents, while one participant (6.67%) had unsuccessfully tried four. Clonazepam (in all formulations and dosages) was the most frequently prescribed drug and had been trialed by all patients, followed by gabapentin (*n* = 4), pregabalin (*n* = 4), and alpha-lipoic acid (*n* = 4). Furthermore, previous prescription histories revealed the presence of Vitamin D (*n* = 2) and Vitamin B (*n* = 1) in the patients’ records.

In relation to the suspension or reduction of current drug prescriptions, 13 participants (86.7%) indicated that they had either suspended (*n* = 8) or reduced (*n* = 5) their medication intake.

### 3.3. Treatment Satisfaction and Likelihood of Recommendation

The results indicated that both treatment satisfaction and the likelihood of recommending the treatment yielded positive outcomes, with an average rating of 7.87 (±2.17) and 8.33 (±2.19), respectively, on an 11-point Likert scale.

### 3.4. Patient Follow-Up

Subsequent to the completion of the study, patients were presented with the option of being discharged from the study or continuing their treatment under the same parameters and conditions. All participants elected to continue their treatment.

No significant clinical or specific BMS medication uptake changes were reported.

## 4. Discussion

An analysis of the results denied the null hypothesis, demonstrating that long-term PBM with LLLT in BMS patients contributed to a significant improvement in HRQoL, both over the course of 6 months and 12 months, as measured by the EQ-5D-5L questionnaire. Moreover, the treatment had a favorable impact on patients’ specific pharmacological profiles and uptake, as evidenced by the successful suspension or reduction of previous BMS therapies, achieving a significant reduction in drug-related side effects and alleviating the economic cost of BMS. These findings support the efficacy of the applied protocol, successfully answering the main research question and representing a significant and innovative contribution to the field, offering new insights and advancing the understanding of the topic.

The incorporation of the ICOP classification and the establishment of more expansive exclusion criteria to reduce confounding factors enabled the selection of a sample that is consistent with the existing literature [1,5,6,12,16], thereby enhancing the robustness and validity of the findings.

The treatment of BMS has presented a considerable challenge over time, resulting in the investigation of an array of pharmacological classes and supplements. Among these, clonazepam appears to be the most well-established option, although the optimal formulation and dosages remain subjects of debate [15,16]. This was the reality observed in our sample, with all participants currently or previously taking this drug, and more than half having already experimented with at least two different classes of drugs.

The intra- and extracellular processes in the mechanism of action of PBM, particularly when combined with LLLT, present biological stimulation properties. These properties have been demonstrated to result in favorable outcomes in the reduction of symptoms and enhancement of BMS patients’ quality of life, with results that exceed those observed in control groups (placebo treatments or pharmacological interventions) [16,17,18,19,20,21,22]. However, Camolesi and colleagues identified a notable trend indicating that red laser protocols appeared to offer more pronounced benefits than other wavelengths [23]. However, these studies relied on PBM sessions a weekly basis, or multiple occasions within the same week, with short-term evaluations at the termination of the trial or not exceeding a few months.

The present study employed Photobiomodulation (PBM) therapy conducted using a low-level laser therapy (LLLT) device operating within the red-light wavelength (λ = 660 nm). Furthermore, the affected areas were irradiated once every two weeks over a period of 12 months, resulting in a highly efficacious outcome. These findings are not only in accordance with those of Camolesi [23] but also substantiate the benefits of long-term PBM therapy in BMS patients, which represents a novel approach in this field.

Patients’ HRQoL demonstrated a statistically significant improvement in four dimensions (self-care, usual activities, pain/discomfort, and anxiety/depression) of the EQ-5D-5L questionnaire. A similar trend was also noted in the EQ-VAS questionnaire, which evaluates patients’ health perception, showing a mean score increase of 45.27 points, which is more than double the initial values. This evidence illustrates that although BMS symptoms are limited to the oral cavity [1,6], they can markedly influence the ability to perform essential activities such as oral hygiene, eating, communication, and social interactions, as well as the patients’ overall perception of their health status. As anticipated, no change was observed in the fifth dimension, which pertains to patients’ mobility. This discovery is consistent with the absence of a correlation between BMS and patient mobility. This is because the former is limited to symptoms within the oral cavity and has no impact on patients’ movement.

The application of the Spearman’s Rho (ρ) test also revealed several statistically significant correlations between all four improved dimensions and the VAS score. These findings are consistent with those reported in the existing literature, which spans various studies, populations, and diseases [26,27,28]. This illustrates the effectiveness of administering the EQ-5D-5L questionnaire to patients with Burning Mouth Syndrome, as it accurately reflects the diversity of their clinical manifestations. Another advantage of utilizing this questionnaire is its simplicity, comprising only five questions and a visual analog scale (VAS), in comparison to other instruments for evaluating QoL or oral health-related QoL, such as the Medical Outcomes Study Short Form Survey-36 (SF-36) [29] or the Oral Health Impact Profile (OHIP) [30], which present a higher level of complexity.

Another notable finding of this study is the improvement in patients’ HRQoL and health perception when comparing the results at 6 months and 12 months. This contributes to the hypothesis previously put forth by Hawkins et al. and Máximo et al. that the effects of PBM with LLLT may be cumulative [31,32].

The level of patient satisfaction and the probability of a recommendation were also found to correlate with the clinical outcomes, with an average rating of 7.87 (±2.17) and 8.33 (±2.19), respectively, on an 11-point Likert scale. These findings indicate that the duration and regularity of the treatment may be a contributing factor to the observed scores.

In light of the aforementioned findings, the presented protocol represents a novel therapeutic option for the treatment of BMS patients. This approach can therefore alleviate patients’ symptoms, improve their HRQoL, and allow for the successful reduction or even suspension of certain specific prescriptions. Furthermore, patients’ high satisfaction with the treatment will yield social, labor, and psychological benefits. Nevertheless, new long-term protocols must present data in order to ascertain the optimal interval between sessions and to establish the correlation between different LLL parameters.

### Limitations

While the results of this study appear promising, several limitations must be considered. While the reduced sample size is consistent with the design of a unicentric clinical study and can impact the statistical analysis, a prospective multicentric collaborative study can be considered. Additionally, the use of self-report questionnaires, which are inherently subjective, may introduce a potential bias.

Furthermore, the long-term consequences of continuous PBM LLLT have yet to be elucidated, engendering ambiguity regarding its capacity to effect a cure and underscoring the necessity for chronic therapy, thereby underscoring the importance of tailored treatment plans for each patient.

## 5. Conclusions

This study presents a distinctive approach to evaluating the use of PBM in BMS patients. It assesses the long-term effects of continuous biweekly therapy on patients’ HRQoL and pharmacologic profile.

The findings indicate that PBM has a significant positive impact on patient HRQOL and effectively addresses the symptoms associated with BMS, thereby reducing or even eliminating the need for previous medications in some cases.

Moreover, the findings offer insight into the efficacy of an outpatient-based PBM with LLLT applied protocol, indicating that the proposed therapeutic approach is well suited for implementation in a routine outpatient setting.

Given the innovative methodology and promising results, further research with larger sample sizes, control groups, and varied LLLT parameters is essential to advance knowledge and optimize treatment protocols in this field.

## Figures and Tables

**Table 1:** Variation analysis of the EQ-5D-5L questionnaire results.

EQ-5D-5L	Friedman Test	df	*p*	Kendall’s W
Mobility	.	2	.	
Self-Care	28.500	2	<0.001 *	0.950
Usual Activities	27.395	2	<0.001 *	0.913
Pain/Discomfort	27.125	2	<0.001 *	0.904
Anxiety/Depression	27.362	2	<0.001 *	0.912
Perceived Health Level	28.429	2	<0.001 *	0.948

* Correlation is significant at the *p* < 0.05 level.

**Table 2:** Correlations between the EQ-5D-5L improved dimensions, perception of health, and treatment satisfaction after 12 months.

			Treatment Satisfaction	EQ-5D-5L Perceived Health Level	EQ-5D-5L Self-Care	EQ-5D-5L Usual Activities	EQ-5D-5L Pain/ Discomfort	EQ-5D-5L Anxiety/ Depression
Spearman’s Rho	Treatment Satisfaction	Correlation Coefficient	1.000	0.609 *	−0.890 **	−0.807 **	−0.781 **	−0.825 **
		Significance (2-tailed)		0.016	0.000	0.000	0.001	0.000
	EQ-5D-5L Perceived Health Level	Correlation Coefficient	0.609 *	1.000	−0.522 *	−0.502	−0.509	−0.598 *
		Significance (2-tailed)	0.016		0.046	0.056	0.053	0.019
	EQ-5D-5L Self-Care	Correlation Coefficient	−0.890 **	−0.522 *	1.000	0.610 *	0.742 **	0.816 **
		Significance (2-tailed)	0.000	0.046		0.016	0.002	0.000
	EQ-5D-5L Usual Activities	Correlation Coefficient	−0.807 **	−0.502	0.610 *	1.000	0.755 **	0.647 **
		Significance (2-tailed)	0.000	0.056	0.016		0.001	0.009
	EQ-5D-5L Pain/ Discomfort	Correlation Coefficient	−0.781 **	−0.509	0.742 **	0.755 **	1.000	0.782 **
		Significance (2-tailed)	0.001	0.053	0.002	0.001		0.001
	EQ-5D-5L Anxiety/ Depression	Correlation Coefficient	−0.825 **	−0.598 *	0.816 **	0.647 **	0.782 **	1.000
		Significance (2-tailed)	0.000	0.019	0.000	0.009	0.001	

* Correlation is significant at the *p* < 0.05 level. ** Correlation is significant at the *p* < 0.01 level.

## Data Availability

The datasets presented in this article are not readily available because they are part of an ongoing study and are protected by national privacy laws.
